# Developing Youth's Capacity to Lead Through Complexities: Exploring 4‐H Extension Agents’ Perception on Barriers and Opportunities

**DOI:** 10.1002/yd.70057

**Published:** 2026-05-15

**Authors:** Israel O. Oyedare, Eric K. Kaufman

**Affiliations:** ^1^ Agricultural, Leadership, and Community Education Virginia Tech Blacksburg Virginia USA

## Abstract

As the world faces dynamic challenges affecting societies and organizations, there has been an increasing call for the development of transformational leadership among youth. This focus is critical for preparing for the future and for inspiring hope, trust, and cooperation. Unfortunately, despite the popularity of the transformational leadership domain, the emphasis has largely been on adult leaders. Thus, this study explores 4‐H Extension agents’ perspectives about barriers to and support areas for fostering transformational leadership development (TLD) among youth. Interviews revealed primary barriers and strategies that influence success with youth TLD, acknowledging the adults can both inhibit and facilitate the process. The findings have important implications for multiple stakeholders, including 4‐H Extension services, community organizations, and academic institutions.

## 1 Introduction

As more wicked and complex problems emerge, developing new approaches to foster transformational leadership skills in youth has become imperative (Andenoro and Skendall 2020; Farahnak et al. 2020). According to Anselmann and Mulder (2020), equipping youth with transformational leadership skills will support them in adapting to the present‐day global dynamics while inspiring hope, trust, and cooperation. Mason et al. (2014) suggested transformational leadership development (TLD) for youth emphasizes the development of both interpersonal and intrapersonal skills, as well as the ability to think transcendentally and solve complex problems. This aligns closely with Bass and Avolio's (1995) assertion that TLD involves developing a personality that inspires and motivates others toward bigger and greater goals. Similarly, Kouzes and Posner (2024) posited exemplary leadership (synonymous to transformational leadership) should focus on strengthening abilities to model the way, inspire a shared vision, challenge the process, enable others to act, and encourage the heart. Consistent with this literature, two central themes are essential: youth's ability to develop a vision for change and to communicate that vision persuasively.

Vision development entails a relational process of identifying what motivates followers and then creatively co‐constructing a shared and compelling vision (Lehmann‐Willenbrock et al. 2015). On the other hand, persuasive communication involves the artistic and charismatic articulation of this shared vision, as well as the ability to influence and motivate followers to work toward its achievement (Williams et al. 2018). Accordingly, TLD emphasizes efforts to build meaningful connections and foster engagement with followers (or peers), thereby increasing followers’ motivation to perform beyond expectations. In today's dynamic world, these skillsets are needed to enable youth to solve complex challenges (Anselmann and Mulder 2020).

Nevertheless, TLD, as an area of research, is predominantly focused on adult leaders with limited emphasis on the youth population (Korejan and Shahbazi 2016). Although certain organizations incorporate elements of transformational leadership within their youth development initiatives, there remains limited empirical evidence demonstrating the impact of TLD on youths’ capacity to address complex challenges. Moreover, most existing studies examine the application of TLD by adult leaders working with youth (Turnnidge and Côté 2018; Newland et al. 2019), rather than exploring how youth enact transformational leadership themselves or influence adults through these practices.

For more than a century, 4‐H Extension has organized and delivered youth development programming, positioning it as the largest youth development organization in the United States (De Guzman and Hatton 2024). County 4‐H Extension agents play a central role in this system by translating research‐based knowledge into locally responsive youth education and programming. These agents are trained to identify community‐level challenges affecting youth, design and implement evidence‐informed strategies, and facilitate experiential learning across domains (Ferrari et al. 2009; Sundgren 2019). Their work is explicitly oriented toward developing youth who are prepared to address current and future societal challenges (Arnold and Gagnon 2020). Accordingly, 4‐H Extension agents develop competencies in community engagement, social action, pedagogy, interpersonal relations, and leadership and curriculum development—positioning them as a critical source of insight into youth‐focused TLD (De Guzman and Hatton 2024).

The purpose of this research project was to explore perceptions of 4‐H Extension agents on barriers to and support areas for youth TLD. Specifically, this study was guided by two questions:

1. What barriers hinder the TLDof 4‐H youth?
2. What support is needed to effectively foster TLD within 4‐H programs?

The answers to these research questions hold the potential for practitioners to advance innovative ideas for developing the transformational leadership skills of youth and helping them to be change agents.

## 2 Leadership Development Begins From Childhood

Children's perception of leadership starts forming through their distinctive exposure and experience with an adult leader playing the role of nurturing and mentorship (Escobar Vega et al. 2024). At this stage, children engage in multiple developmental strategies, including shared activities, social learning, and discovery that influence their implicit ideology about leadership (Eden & Leviatan, 1975). These mental models—often called *schema*, *prototype*, or *expectation*—affect how people judge leadership behaviors and performance (Ayman‐Nolley and Ayman 2005). These dynamic experiences develop into categories through which perceptions, memories, and interaction with leadership are formed (Lord et al. 2020).

One commonality between adults and children is their leadership orientation toward task and relationship (Ayman‐Nolley and Ayman 2005). Accordingly, children's leadership development is significantly contextual based on exposure. Moreover, humans naturally have intrinsic helping behaviors without expectations for rewards and consideration, with childrens’ innate socialization and feedback from interactions playing a crucial role in shaping altruistic behaviors as they grow into adolescents (Warneken and Tomasello 2009). Notably, children who witness significant adults acting altruistically and doing it enthusiastically have the tendency to grow genuine altruism as a motivational force (Goldstein & Brooks, 2021).

## 3 Trends in Youth Engagement With 4‐H Programs

Over the years, 4‐H has designed programs and structures for engaging youth and empowering them to participate in their community and make a difference in real situations (Rosser et al. 2008). In implementing these programs, the 4‐H Thriving Model was designed to foster youth participation in high‐quality 4‐H programs and highlight the process of positive youth development within the organization (Arnold and Gagnon 2020). The Thriving Model emphasizes seven key indicators: openness to challenge and discovery, growth mindset, hopeful purpose, prosocial orientation, transcendent awareness, positive emotionality, and self‐regulation through goal setting and management (4‐H, n.d.). While the thriving model has been broadly adopted (Elliot‐Engel et al. 2018; Go‐Maro 2024), intermediate and senior level 4‐H members (ages 12–18) tend to be less committed to these programs as they grow into various stages of life (Albright 2008). Although many studies have explored the rationale behind the drop in enrollment and interests in 4‐H programs (see Albright and Ferrari 2010; Ellison and Harder 2018), few studies focus on targeted programs or initiatives.

## 4 Theoretical and Conceptual Framework

This study is grounded in path–goal theory, which emphasizes the interactive relationship between followers and goal attainment (House 1971; 1996). The theory highlights leaders’ roles in defining goals, clarifying pathways, removing obstacles, and providing support needed for success (Northouse 2022). While 4‐H youth feel confident about their future careers, they also recognize the need for stronger life and leadership skills to create societal impact (Nwoko 2024). Consistent with this, 4‐H prioritizes skills such as collaboration, communication, and interpersonal competence over technical skills, helping prepare youth to be “beyond ready” for future challenges (Hart Research 2024). The “Beyond Ready” initiative reflects 4‐H's commitment to complementing formal education by building youth resilience, confidence, and capacity for community and global impact (Bramble, 2024).

These goals align with the Bass and Riggio's (2005) assertion that TLD is not achieved accidentally but through a long‐term process of exposure to high degree of moral standards, engagement in leadership activities over time, and formation of personal ideals and conviction to be other‐centric (i.e., showing concern for others and for society). This further involves building key personal, emotional, and inspirational skills required to develop and articulate a vision, and mobilize followers to achieve the same (Nica 2014). The development of these transformational attributes among youth requires systematically examining the perceptions of youth leaders regarding barriers and support areas for youth TLD. As asserted by Bans‐Akutey (2021), path‐goal theory provides the opportunity to contextualize experiences and situations. In the case of TLD, the path‐goal theory captures the systematic process of goal setting, resource mobilization, and constraint determination.

**FIGURE 1:**
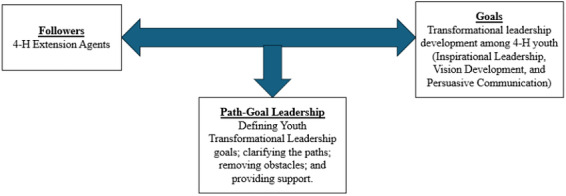
Interaction between youth transformational leadership development and the path‐goal theory. Note. A simplified model that illustrates the relationship between youth transformational leadership development and the path‐goal theory for the purpose of this study.

Therefore, and as seen in Figure 1, this study adopts path‐goal theory, by framing 4‐H Extension leaders as goal‐setters guiding youth toward TLD. Extension agents serve as followers and study participants, while the goal is the TLD of 4‐H youth. The focus of this study is in addressing two of the paths (i.e. addressing barriers and support areas) that lead to the fulfilment of the goal.

## 5 Methods

This study employed a qualitative research design to explore 4‐H Extension agents’ perceptions of barriers and strategies for fostering TLD among youth. According to Crabtree and Miller (2023), the qualitative method provides opportunity for engaging in interpretative, focused, and natural activity with the goal of receiving a holistic and descriptive explanation. This study used snowball and convenience sampling methods to recruit 4‐H Extension agents from the state 4‐H Extension, the Southern Region Program Leadership Network 4‐H Committee, and the National 4‐H (through the USDA). Participants were recruited based on interest and further referrals from initial respondents, allowing access to a wider network within the 4‐H community.

Following Hennink and Kaiser's (2022) guideline that 9 to 17 interviews is typically sufficient to reach saturation in qualitative research; this study interviewed 15 Extension agents affiliated with 4‐H. Details on each are provided in Table 1.

**TABLE 1:** Demographics of 4‐H extension agents interviewed for the study.

Pseudonym	4‐H Region	Length of Service	4‐H Background?
Angela	Southern	5 years	Yes
Bernard	Northeast	32 years	No
Blake	Northeast	15 years	Yes
Claire	North Central	12 years	Yes
Daniella	Southern	11 years	No
Faith	Southern	9 years	No
Gabriella	Northeast	4 years	Yes
Idris	Southern	6 years	Yes
Joel	Southern	20 years	Yes
Kate	Northeast	9 years	No
Kasie	Northeast	8 years	No
Luke	Southern	32 years	Yes
Mary	Southern	2 years	No
Nasir	Southern	12 years	No
Ruth	Western	20 years	Yes

*Note*. For the 4‐H Background, “Yes” indicates the participant was previously a 4‐H member and “No” indicates they were not a 4‐H member.

Qualitative data were collected through semi‐structured interviews, where questions were phrased to help participants reflect on the two transformational leadership components—vision development and persuasive communication. After data collection, interviews were transcribed, cleaned, and anonymized. A line‐by‐line analysis was conducted in three steps: transcripts were reviewed for clarity, excerpts were analyzed for meaning, and data were coded and organized thematically using a combination of Atlas.ti and Microsoft Excel.

To ensure trustworthiness during data collection, the interview guide was pilot tested with 4‐H Extension leaders and underwent multiple rounds of review by two leadership educators, one of whom had decades of experience as an Extension specialist. Following the interviews, transcripts were shared with participants for review and feedback. Additionally, transparency in the analysis phase was enhanced through rigorous and iterative reviews conducted by the researcher, alongside oversight from a committee of advisors and reviewers with extensive expertise in qualitative research. This committee provided critical feedback on both the analytical and methodological processes.

## 6 Results/Findings

Findings highlighted how 4‐H Extension agents perceive barriers to and strategies to support youth in TLD. Through analysis of participant experiences, several themes emerged that explicitly answer the two research questions.

### 6.1 Research Question 1: What Barriers Hinder the TLD of 4‐H Youth?

From the data collected and analyzed, the findings reveal that structural barriers, inadequate support, youth‐focused barriers, and programmatic limitations collectively hinder TLD among 4‐H youth. While these barriers compound to affect youth's ability to develop transformational leadership skills, participants emphasized adult's reluctance to share power and provide youth with little or no meaningful control as a major barrier.

#### 6.1.1 Structural Barriers

In directly addressing the first research question, participants expressed how structural barriers in part inhibited TLD among 4‐H youth. They shared how 4‐H is a complex organization with more focus on rural youth than urban youth. Example emergent codes include: “4‐H can be a complex organization,” “focused more on rural youth than urban youth,” and “4‐H tries to engage in multiple programs that reduce its quality.” Relatedly, Claire echoed her frustration about the structure of 4‐H, noting the complexity of 4‐H lessens the value of TLD program:

In our program, 4‐H can kind of be a complex organization, and the reality is you can do anything in 4‐H, which is really overwhelming. As Extension agents, we spread ourselves too thin and try to be all things to every youth and community; sometimes I think it lessens the quality of the programs we are able to provide.

Luke further expressed: “I am worried there are only so many 4‐H‐ers that are going to go into high school from these clubs.” Despite many 4‐H youth not being enrolled in high school, Nasir believed that “in many states and localities, 4‐H is limited to high school,” with little or no presence at the college level. This barrier makes it impossible for youth to apply the transformational leadership skills garnered in a formal setting and to interact with people beyond their immediate environments.

Overall, the participants shared that structural barriers such as unequal access to 4‐H programs and complexity of the 4‐H organization are barriers to TLD among intermediate and senior 4‐H youth.

#### 6.1.2 Inadequate Support System

Further providing response to the barriers to the TLD of 4‐H youth, participants shared how common it was to see adult leaders who are unwilling or lack the capacity to give up leadership for the youth to lead and acquire skills necessary for transformational leadership. The data collected reflected codes such as “Adults’ inability to give up power to youth,” “Adults that do not believe in their youth,” and “Few trained Extension agents that can cater for 4‐H youth.” One of the research participants, Idris, stated: “If youth don't have a leader that is able to step back and allow the youth to actually grow and develop, that's probably their biggest restraint.” Kate also mentioned that “it can be hard for the adult to let go and see the value in letting go of their power.” Moreover, Bernard shared that youth need more support from adults:

The guardians or parents of the youth are not providing guidelines, and you know how much intermediate and senior youth can handle on their plate…. With both parents working outside of the home, youth may not get enough time with their mentor or parent and may lack the necessary resources and support to accomplish their goals.

Some guardians lack modern skills to support their youth. Faith observed: “We also have a lot of grandparents, who have very limited digital literacy, that are raising these kids.” Nasir highlighted time constraints and noted that many “youth feel like they don't have a strong support system.”

This finding of “Inadequate Support System” as a barrier demonstrates that while TLD may be targeted at the youth, the unwillingness and limited capacity of adults to enable such development can fundamentally hinder its realization.

#### 6.1.3 Youth‐Focused Barriers

Further acknowledging barriers, participants shared that youth perceptions may influence their commitment to and enrollment for TLD programs. These perceptions may be “a result of past experiences of the youth” (Kasie). Also, the area where the youth are from influences their perceptions toward TLD. Angela conveyed this in her statement:

The youth that I work with on the state 4‐H cabinet were really struggling with how they felt they were being treated because of where they lived. In general, living in the Southern region has a lot of stereotypes associated with not being intelligent—there are a lot of challenges that come with that—and so the youth kind of felt like they were being looked down upon.

Other participants reported that youth fear their ideas may not be accepted: “the youth are very timid” (Luke), “sometimes, they can be intimidated” (Blake), “it's more that they are afraid” (Idris). Moreover, youth have a variety of external demands, including time demands and commitments to high school sports, personal and relational demands: “Youth get into high school; and it's basketball, baseball, football, and soccer, and everything else in the world” (Luke). Nasir shared, “Many of the youth that I work with have to have jobs in order to sustain their household or even themselves.”

This finding of “Youth‐Focused Barriers” underscores that 4‐H youth are often faced with conflicting demands and experiences that influence their perception and commitment to the TLD process.

#### 6.1.4 Programmatic Barriers

Lastly, participants shared how some of the learning processes adopted by 4‐H may not be sustainable enough for 4‐H youth to practice. For instance, Claire lamented about the lack of a plan for keeping the youth: “We need to clarify what long‐term engagement in a program can look like for youth. We do a good job of recruiting youth for a one‐time program, but how can we continue to engage them and build from those experiences?” Many of the activities “may not be too challenging for the youth” (Mary) or “are not too cool for the youth to attract their participation” (Joel). Nasir lamented that “a lot of youth are not introduced to 4‐H TLD programming.”

Transportation and distance issues sometimes hinder participation. Idris mentioned, “Well, every youth is not going to come out on a Tuesday in my county, because depending upon where they live, traffic is wild and it could take them an hour and a half to get there.” Faith shared similar thoughts:

This barrier does happen on a local level, and it is transportation for youth to get to the location for them to practice leadership skills. I have plenty of youth that would love to be a part of more 4‐H things, and I can try to offer it in the most centrally located spot in my locality, but they still might have to drive 30 to 45 min and that's kind of challenging for them.

In sum, participants communicated that TLD initiatives within the 4‐H lack youth‐friendly approaches and considerations.

### 6.2 Research Question 2: What Support Is Needed to Effectively Foster TLD Within 4‐H Programs?

Regarding supports needed for the TLD of 4‐H youth, Extension agents called for more focus on capacity building for adults, partnership between adults and youth, providing more opportunities for youth to solve complex problems, and creating more effective TLD program. Imperatively, the findings revealed that TLD can be achieved through collaborative efforts among organizations, adults, and youth.

#### 6.2.1 Developing the Capacity of Adult Leaders in the Area of Transformational Leadership

As a first strategy for fostering TLD among youth, participants mentioned an effective approach for supporting the TLD of 4‐H youth is by developing the capacity of adult leaders for transformational leadership. This involves training and educating adult leaders on conceptual and practical approaches. Kate shared: “So many of the programs that I have been able to attend as an agent, I come back with a beautiful manual to adopt in the communities where I serve, specifically for youth‐related programs.” Luke reinforced the point:

I think we need to focus a little more on training for our adults, some of whom are volunteers. We need to think about doing a little more for our volunteers and show them that the youth are really looking up to you. Adults need to shepherd the youth in the right direction by making sure that our volunteers are trained, and that they understand their role as teachers and educators. I think a strategy for 4‐H is to be more mindful of the fact that we need our volunteering adults to understand that they are instructors and teachers, and that you give them not just a lesson plan but guidelines in plain languages.

This finding reflects the need for an equal focus on the TLD of adults who engage with youth. As identified during the data collection process, many Extension agents expressed appreciation for training opportunities provided by Extension and reported observable changes in their practices as a result of participation.

#### 6.2.2 Enabling Youth's Ability to Solve Complex Problems

Furthermore, participants expressed that a great strategy for supporting the TLD of 4‐H youth is by enabling their ability to solve complex problems through provision of opportunities for youth to explore novel ideas and to garner change‐making skills. Mary stated that “club volunteers, who are adult leaders, should really focus on what the youth can do at their current stage and encourage them to chase their dreams.” Similarly, Kasie shared that “we can support youth by giving them authentic experiences in 4‐H—not just talking about it but actually putting the youth in leadership roles.” Moreover, Blake emphasized a pragmatic approach:

When our communities are looking for youth participation, encourage the youth to step up. We have town meetings here in our county where the community leaders decide on specific issues… Put the youth at the table and engage them in the decision‐making process so that they can model positive behavior.

Adult leaders will need to play active roles of “really helping those young people narrow in on what it is they are trying to accomplish,” Clair suggested. Kate further supports that “empowering the youth to set those SMART goals with timeline and expectation is key. The job of an adult leader is to kind of poke holes.” Ultimately, Faith mentioned that “having things more localized for our youth to participate in these leadership opportunities would be great and will increase transformational leadership for [them].”

This strategic finding underscores that transformational leadership skills are built when youth are provided opportunities to participate in changemaking processes. As identified by participants, youth learn skills such as vision development, communication, and problem solving, which are critical areas of transformational leadership.

#### 6.2.3 Building Effective TLD Programs

Thirdly, participants conveyed a need to make TLD programs more appealing to youth and accessible across multiple communities. For example, Idris said:

Being more intentional in the way we market our programs and where we market them is important. For instance, when an adult leader goes to a community where the majority of the youth are in the Muslim faith, the youth there may not want to participate in 4‐H programs, because they might not know what it means. However, it is the duty of the Extension agent to find a trusted adult within that community and build a relationship with the adult such that they can vouch and recommend youth to participate.

According to Luke, “if the youth find the transformational leadership program fun, they are going to come back with the feeling that they are a part of something huge.” Similarly, Joel shared that programs should be implemented in a way that the youth “will buy‐in and feel comfortable with the program.” Idris recommended “do[ing] a think tank that gives the youth opportunities to just throw out their ideas and wildest dreams and see if it can turn into fruition.”

Imperatively, Mary highlighted the importance of flexibility: “Adult leadership should build a club that aligns with youth's family values. As a matter of fact, I have several clubs that meet on Monday nights because they have devout religious families that won't meet on the other nights.”

This strategic finding underscores that TLD among 4‐H youth is contingent on effective programming, supported by adequate financial and non‐financial resources, intentional planning, and youth‐friendly engagements.

#### 6.2.4 Youth‐Adult Partnership

Lastly, participants considered youth‐adult partnership a great strategy for supporting TLD. Kasie shared that adults need to create an incremental relationship with the youth: “It's about creating a developmental relationship with the youth. Adults need to be aware and intentional about creating that relationship, so that the youth feel safe and can feel like their voice can be heard.” To effectively collaborate with the youth, adults need to connect with them. Angela said:

That's our future, and that's the only way we are ever going to make change, which is by connecting with those youth who are in that integral stage of life, and preparing to become the future leaders of the world… I think this affords the youth opportunity to have a deeper connection with a caring adult mentor. The goal is to help the youth feel comfortable sharing their problems and asking advice from adult mentors.

Furthering the focus on “partnership,” Claire recommended adult leaders “guide and support the youth without taking over the leadership role.” Blake shared “that adult leaders [should] act as a sounding board for the young people who want to engage with the adults.” In addition, Mary reported that “adult leaders have to step back and let the youth lead.”

Overall, this strategy finding emphasizes the collaborative role that can exist between adults and youth in TLD, highlighting that greater progress is achieved when adults actively partner with youth in their growth.

## 7 Discussion/Recommendations/Conclusion

Drawing from 4‐H Extension agents’ perceptions, findings revealed four primary barriers to youth TLD: structural, inadequate support systems, youth‐focused, and programmatic. Structurally, tensions between 4‐H's rural legacy and its mission to serve diverse populations, coupled with limited adaptation to digital engagement, limit the organization's capacity to develop comprehensive TLD efforts (Elliott‐Engel et al. 2024; Jensen et al. 2021). Participants also highlighted adult reluctance to share leadership and decision‐making power with youth, which limits opportunities for youth to develop vision and collaborative leadership skills (Welton et al., 2021) and may reduce youths’ trust in adult leaders (Redmond and Dolan 2016).

Research by Conner et al. (2016) further suggested how adults may, at times, constrain youths’ potential by discouraging or undermining their pursuit of transformational leadership. Adults’ perceptions, prior experiences, and motivations can shape how power is distributed, often resulting in unequal dynamics that restrict youths’ autonomy, limit their ability to explore their skills, and hinder their active engagement in social change (Corney et al. 2021; Schusler et al. 2017). Moreover, youth‐focused barriers included perceptions, personality traits, and external demands that shape engagement in leadership initiatives. Beliefs formed through past experiences can influence youths’ commitment to leadership development (Schyns et al. 2011). Programmatic barriers such as insufficient funding, lack of long‐term planning, and organizational constraints further restrict participation (Agans et al. 2020).

Turning to an opportunity focus, findings revealed four strategies to support youth TLD. First, adult leaders must intentionally learn and model transformational leadership. Although 4‐H Extension agents receive training in youth development and program delivery (Sundgren 2019), tailored transformational leadership training is needed for all adults working with intermediate and senior 4‐H youth, including volunteers. Second, enabling youth to address complex societal problems strengthens transformational leadership skills and promotes grassroots community development (du Plessis et al. 2024). This process should include youth participation in decision‐making at local, sub‐national, and national levels (Augsberger et al. 2024). Third, findings highlight the importance of collaboratively designing culturally and community‐responsive leadership programs with youth to enhance accessibility and sustained engagement (Khan 2022; Song and Hur 2022). Finally, strong youth–adult partnerships were identified as essential for leadership development, fostering communication, cognitive, and emotional growth through collaboration and shared decision‐making (Kokozos and Gonzalez 2024).

### 7.1 Implication for Leadership Education and Practice

In line with the study's recommendation to create opportunities for youth to explore innovative ideas and cultivate change‐making skills, leadership educators can integrate experiential learning activities—such as group and individual assignments, as well as service‐learning projects—into leadership curricula. These practices not only reinforce theoretical knowledge but also provide practical contexts for students to develop and demonstrate transformational leadership behaviors. Moreover, findings from this research suggests that leadership educators design activities that are appealing to youth and reflect their diverse situational experiences through robust youth‐learning activities (Allen et al. 2022). This approach empowers youth by fostering interest in the learning process, promoting self‐directed learning, and cultivating a supportive and engaging learning environment (Johnson 2014).

Additionally, youth development organizations can use these findings to strengthen strategies that prepare youth for transformational leadership in communities and organizations. There is a persistent gap in the transfer of leadership knowledge and skills from adults to youth, limiting effective succession planning (Megheirkouni and Mejheirkouni 2020). This study indicates that closing this gap requires intentional, practice‐oriented leadership development approaches that move beyond traditional adult‐centered models. Similarly, there is a need for structured youth–adult partnerships in which adults serve as collaborators, mentors, and facilitators rather than sole decision‐makers. By sharing power, fostering trust, and modeling leadership behaviors, adults can support youths’ TLD (Seymour 2017). For example, providing youth with autonomy to lead innovative projects, paired with developmentally appropriate guidance, helps cultivate competencies such as vision, motivation, and communication (Thompson and Vecchio 2009). Additionally, investing in adult leaders’ TLD may enhance their capacity to model effective leadership and create environments that support youth growth (Robertson et al. 2021).

## References

[yd70057-bib-0002] Agans, J. P. , M. Maley , N. Rainone , et al. 2020. “Evaluating the Evidence for Youth Outcomes in 4‐H: A Scoping Review.” Children and Youth Services Review 108: 104617. https://open.clemson.edu/joe/vol52/iss4/35.

[yd70057-bib-0003] Albright, M. B. 2008. Here Today, Gone Tomorrow: An Investigation Into Why Older Youth Leave the 4‐H Program, [Doctoral dissertation]. The Ohio State University.

[yd70057-bib-0004] Albright, M. B. , and T. M. Ferrari . 2010. ““Push” and “Pull” a Qualitative Study of Factors That Contribute to Older Youth Leaving the 4‐H Program.” Journal of Youth Development 5, no. 3: 55–74. 10.5195/jyd.2010.209.

[yd70057-bib-0005] Allen, S. J. , D. M. Rosch , and R. E. Riggio . 2022. “Advancing Leadership Education and Development: Integrating Adult Learning Theory.” Journal of Management Education 46, no. 2: 252–283. 10.1177/10525629211008645.

[yd70057-bib-0006] Andenoro, A. C. , and K. C. Skendall . 2020. “The National Leadership Education Research Agenda 2020–2025: Advancing the State of Leadership Education Scholarship.” Journal of Leadership Studies 14, no. 3: 33–38. 10.1002/jls.21714.

[yd70057-bib-0007] Anselmann, V. , and R. H. Mulder . 2020. “Transformational Leadership, Knowledge Sharing and Reflection, and Work Teams' Performance: A Structural Equation Modeling Analysis.” Journal of Nursing Management 28, no. 7: 1627–1634. 10.1111/jonm.13118. 32754940

[yd70057-bib-0008] Arnold, M. E. , and R. J. Gagnon . 2020. “Positive Youth Development Theory in Practice: An Update on the 4‐H Thriving Model.” Journal of Youth Development 15, no. 6: 1–23. 10.5195/jyd.2020.954.

[yd70057-bib-0009] Augsberger, A. , M. E. Collins , and R. C. Howard . 2024. “The Global Context of Youth Engagement: A Scoping Review of Youth Councils in Municipal Government.” Children and Youth Services Review 156: 107349. 10.1016/j.childyouth.2023.107349.

[yd70057-bib-0010] Ayman‐Nolley, S. , and R. Ayman . 2005. “Children's Implicit Theory of leadership.” In Implicit Leadership Theories: Essays and Explorations, edited by B. Schyns and J. R. Meindl , 189–233. Information Age Publishing.

[yd70057-bib-0011] Bans‐Akutey, A. 2021. The path‐goal theory of leadership. Academia Letters, Article 748. 10.20935/AL748.

[yd70057-bib-0012] Bass, B. M. , and B. J. Avolio . 1995. MLQ multifactor leadership questionnaire for research. Mind Garden.

[yd70057-bib-0058] Bass, B. M. , and R. E. Riggio . 2005. “The Development of Transformational leadership.” In Transformational Leadership, edited by B.M. Bass and R.E. Riggio (Eds.), (2nd ed., 142–166). Psychology press.

[yd70057-bib-0057] Bramble . (2024. October 7). 4‐H Answers the Call to Building a Ready Generation . 4‐H. https://4‐h.org/about/blog/4‐h‐answers‐the‐call‐to‐building‐a‐ready‐generation/.

[yd70057-bib-0013] Conner, J. O. , C. N. Ober , and A. S. Brown . 2016. “The Politics of Paternalism: Adult and Youth Perspectives on Youth Voice in Public Policy.” Teachers College Record 118, no. 8: 1–48. 10.1177/016146811611800805.

[yd70057-bib-0014] Corney, T. , T. Cooper , H. Shier , and H. Williamson . 2021. “Youth Participation: Adultism, Human Rights and Professional Youth Work.” Children & Society 36, no. 4: 677–690. 10.1111/chso.12526.

[yd70057-bib-0059] Crabtree, B. F. , and W. L. Miller . 2023. Doing Qualitative Research. Sage publications.

[yd70057-bib-0015] De Guzman, M. R. T. , and H. Hatton (Eds.). 2024. Extension Education and the Social Sciences: Uplifting Children, Youth, Families, and Communities. Cambridge University Press.

[yd70057-bib-0016] du Plessis, C. , A. Osman , S. Shakwane , and S. Mokoboto‐Zwane . 2024. “Empowering Youth in Solving Community‐Based Problems: Evidence From South African Schools.” In The Palgrave Handbook of Global Social Problems, edited by R. Baikady , S.M. Sajid , J. Przeperski , V. Nadesan , M.R Islam , and J. Gao , 1–21. Springer International Publishing. 10.1007/978-3-030-68127-2_453-1.

[yd70057-bib-0056] Eden, D. , and U. Leviatan . 1975. “Implicit Leadership Theory as a Determinant of the Factor Structure Underlying Supervisory Behavior Scales.” Journal of Applied Psychology 60, no. 6: 736–741. 10.1037/0021-9010.60.6.736. 1194175

[yd70057-bib-0017] Elliot‐Engel, J. , K. Niewolny , and D. Westfall‐Rudd . 2018. “A Critical Exploration of the Transformative Discourse of 4‐H Youth Development in International Contexts.” In Conference Proceedings: 34th Annual Conference of AIAEE 8–10. https://aiaee.org/PastConferences.

[yd70057-bib-0018] Elliott‐Engel, J. , D. M. Westfall‐Rudd , M. Seibel , E. Kaufman , and R. Radhakrishna . 2024. “Administrators' Perspectives on Organizational Environmental Factors Facing 4‐H Youth Development.” Children and Youth Services Review 156: 107358. 10.1016/j.childyouth.2023.107358.

[yd70057-bib-0019] Ellison, S. , and A. Harder . 2018. “Factors Contributing to the Retention of Senior 4‐H Members: From the Youth Perspective.” Journal of Human Sciences and Extension 6, no. 3: 10. 10.54718/LCJZ7328.

[yd70057-bib-0020] Escobar Vega, C. , J. Billsberry , J. Molineux , and K. B. Lowe . 2024. “The Development of Implicit Leadership Theories During Childhood: A Reconceptualization Through the Lens of Overlapping Waves Theory.” Psychological Review 132, no. 3: 719–743. 10.1037/rev0000484. 38722599

[yd70057-bib-0021] Farahnak, L. R. , M. G. Ehrhart , E. M. Torres , and G. A. Aarons . 2020. “The Influence of Transformational Leadership and Leader Attitudes on Subordinate Attitudes and Implementation Success.” Journal of Leadership & Organizational Studies 27, no. 1: 98–111. 10.1177/1548051818824529.

[yd70057-bib-0022] Ferrari, T. M. , K. S. Lekies , and N. Arnett . 2009. “Opportunities Matter: Exploring Youth's Perspectives on Their Long‐Term Participation in an Urban 4‐H Youth Development Program.” Journal of Youth Development 4, no. 3: 090403FA001. 10.5195/jyd.2009.249.

[yd70057-bib-0023] Go‐Maro, M. 2024. Positive Youth Development (PYD) in Africa: a Case Study of 4‐H Programming in Ghana and Liberia, [Master's thesis, Penn State University]. https://etda.libraries.psu.edu/catalog/32782mvg5315.

[yd70057-bib-0024] Goldstein, S. , and R. B. Brooks . 2021. “Genuine Altruism.” In Tenacity in Children: Nurturing the Seven Instincts for Lifetime Success, (83–93). Springer Nature Switzerland. 10.1007/978-3-030-65089-6_7.

[yd70057-bib-0025] Hart Research . 2024. Beyond ready youth survey [PowerPoint slides]. 4‐h.org. https://4‐h.org/wp‐content/uploads/2024/10/11153116/FINAL‐4‐H‐Beyond‐Ready‐Youth‐Survey‐Deck‐October‐2024‐1.pdf.

[yd70057-bib-0060] Hennink, M. , and B. N. Kaiser . 2022. “Sample Sizes for Saturation in Qualitative Research: Asystematic Review of Empirical Tests.” Social Science & Medicine 292: Article 114523. 10.1016/j.socscimed.2021.114523. 34785096

[yd70057-bib-0026] House, R. J. 1971. “A Path Goal Theory of Leader Effectiveness.” Administrative Science Quarterly 16, no. 3: 321–339. https://www.jstor.org/stable/2391905.

[yd70057-bib-0027] House, R. J. 1996. “Path‐Goal Theory of Leadership: Lessons, Legacy, and a Reformulated Theory.” The Leadership Quarterly 7, no. 3: 323–352. 10.1016/S1048-9843(96)90024-7.

[yd70057-bib-0028] Jensen, M. , M. J. George , M. A. Russell , M. A. Lippold , and C. L. Odgers . 2021. “Does Adolescent Digital Technology Use Detract From the Parent–Adolescent Relationship?” Journal of Research on Adolescence 31, no. 2: 469–481. 10.1111/jora.12618. 33829598 PMC8166296

[yd70057-bib-0029] Johnson, A. P. 2014. “Humanistic Learning Theory.” In Education Psychology: Theories of Learning and human Development. National Social Science Press.

[yd70057-bib-0030] Khan, R. 2022. “Beyond Empowerment and Inspiration: Towards a Critical Program for Multicultural Youth Leadership.” Journal of Youth Studies 25, no. 9: 1284–1300. 10.1080/13676261.2021.1948980.

[yd70057-bib-0031] Kokozos, M. , and M. Gonzalez . 2024. “Collaborating for Social Change: Promising Practices for Effective Youth‐Adult Partnerships.” Journal of Extension 62, no. 3: 33. https://open.clemson.edu/joe/vol62/iss3/33.

[yd70057-bib-0032] Korejan, M. M. , and H. Shahbazi . 2016. “An Analysis of the Transformational Leadership Theory.” Journal of Fundamental and Applied Sciences 8, no. 3: 452–461. 10.4314/jfas.v8i3s.192.

[yd70057-bib-0033] Kouzes, J. M. , and B. Z. Posner . 2024. The Student Leadership Challenge: Five Practices for Becoming an Exemplary Leader, (4th ed.). Jossey‐Bass.

[yd70057-bib-0034] Lehmann‐Willenbrock, N. , A. L. Meinecke , J. Rowold , and S. Kauffeld . 2015. “How Transformational Leadership Works During Team Interactions: A Behavioral Process Analysis.” The Leadership Quarterly 26, no. 6: 1017–1033. 10.1016/j.leaqua.2015.07.003.

[yd70057-bib-0035] Lord, R. G. , O. Epitropaki , R. J. Foti , and T. K. Hansbrough . 2020. “Implicit Leadership Theories, Implicit Followership Theories, and Dynamic Processing of Leadership Information.” Annual Review of Organizational Psychology and Organizational Behavior 7: 49–74. 10.1146/annurev-orgpsych-012119-045434.

[yd70057-bib-0036] Mason, C. , M. Griffin , and S. Parker . 2014. “Transformational Leadership Development: Connecting Psychological and Behavioral Change.” Leadership & Organization Development Journal 35, no. 3: 174–194. 10.1108/LODJ-05-2012-0063.

[yd70057-bib-0037] Megheirkouni, M. , and A. Mejheirkouni . 2020. “Leadership Development Trends and Challenges in the Twenty‐First Century: Rethinking the Priorities.” Journal of Management Development 39, no. 1: 97–124. 10.1108/JMD-04-2019-0114.

[yd70057-bib-0038] Newland, A. , M. Newton , E. W. G. Moore , and W. E. Legg . 2019. “Transformational Leadership and Positive Youth Development in Basketball.” International Sport Coaching Journal 6, no. 1: 1–12. 10.1123/iscj.2018-0002.

[yd70057-bib-0039] Nica, E. 2014. “The Development of Transformational Leadership.” Journal of Self‐governance and Management Economics 2, no. 2: 26–31.

[yd70057-bib-0040] Northouse, P. G. 2022. Leadership: Theory and Practice, (9th ed.). Sage Publications.

[yd70057-bib-0041] Nwoko, H. 2024. 4 in 5 teens believe they will achieve their life goals — But wish they had more technical skills post‐high school. Parents. https://www.parents.com/teens‐believe‐they‐can‐achieve‐their‐life‐goals‐8727518.

[yd70057-bib-0042] Redmond, S. , and P. Dolan . 2016. “Towards a Conceptual Model of Youth Leadership Development.” Child & Family Social Work 21, no. 3: 261–271. 10.1111/cfs.12146.

[yd70057-bib-0043] Robertson, M. N. , H. L. DeShong , J. K. S. Steen , D. R. Buys , and M. R. Nadorff . 2021. “Mental Health First Aid Training for Extension Agents in Rural Communities.” Suicide and Life‐ Threatening Behavior 51, no. 2: 301–307. 10.1111/sltb.12705. 33876485

[yd70057-bib-0044] Rosser, M. , N. L. P. Stedman , C. Elbert , and T. Rutherford . 2008. “Making a Difference: Two Case Studies Describing the Impact of a Capstone Leadership Education Experience Provided Through a National Youth Leadership Training Program.” Journal of Leadership Education 7, no. 3: 84–99. 10.12806/V7/I3/RF1.

[yd70057-bib-0045] Schusler, T. M. , M. E. Krasny , and D. J. Decker . 2017. “The Autonomy‐Authority Duality of Shared Decision‐Making in Youth Environmental Action.” Environmental Education Research 23, no. 4: 533–552. 10.1080/13504622.2016.1144174.

[yd70057-bib-0046] Schyns, B. , T. Kiefer , R. Kerschreiter , and A. Tymon . 2011. “Teaching Implicit Leadership Theories to Develop Leaders and Leadership: How and Why It Can Make a Difference.” Academy of Management Learning & Education 10, no. 3: 397–408. 10.5465/amle.2010.0015.

[yd70057-bib-0047] Seymour, K. 2017. “Building on Strengths: A New Framework for Positive Youth Development Program Practice.” Queensland Review 24, no. 1: 5–22. 10.1017/qre.2017.3.

[yd70057-bib-0048] Song, A. , and J. W. Hur . 2022. “Development of Youth Leadership Through Community‐Based Participatory Action Research During the Covid‐19 Pandemic: A Case Study of Korean American Adolescents.” Journal of Adolescent Research 39, no. 4: 888–916. 10.1177/07435584221078193.

[yd70057-bib-0049] Sundgren, K. 2019. “Feeding Victory: 4‐H, Extension, and the World War II Food Effort.” Online Journal of Rural Research & Policy 14, no. 3: 1–25. 10.4148/1936-0487.1098.

[yd70057-bib-0050] Thompson, G. , and R. P. Vecchio . 2009. “Situational Leadership Theory: A Test of Three Versions.” The Leadership Quarterly 20, no. 5: 837–848. 10.1016/j.leaqua.2009.06.014.

[yd70057-bib-0051] Turnnidge, J. , and J. Côté . 2018. “Applying Transformational Leadership Theory to Coaching Research in Youth Sport: A Systematic Literature Review.” International Journal of Sport and Exercise Psychology 16, no. 3: 327–342. 10.1080/1612197X.2016.1189948.

[yd70057-bib-0052] Warneken, F. , and M. Tomasello . 2009. “The Roots of Human Altruism.” British Journal of Psychology 100, no. 3: 455–471. 10.1348/000712608x379061. 19063815

[yd70057-bib-0053] Welton, A. D. , K. C. Mansfield , and J. D. Salisbury . 2021. “The Politics of Student Voice: The Power and Potential of Students as Policy Actors.” Educational Policy 36, no. 1: 3–18. 10.1177/08959048211059718.

[yd70057-bib-0054] Williams Jr, R. , D. M. Raffo , and L. A. Clark . 2018. “Charisma as an Attribute of Transformational Leaders: What About Credibility?” Journal of Management Development 37, no. 6: 512–524. 10.1108/JMD-03-2018-0088.

